# Influenza vaccination coverage of healthcare workers and residents and their determinants in nursing homes for elderly people in France: a cross-sectional survey

**DOI:** 10.1186/1471-2458-10-159

**Published:** 2010-03-25

**Authors:** Sophie Vaux, Delphine Noël, Laure Fonteneau, Jean-Paul Guthmann, Daniel Lévy-Bruhl

**Affiliations:** 1 Department of infectious diseases. Institut de Veille Sanitaire (InVS) (French Institute for Public Health Surveillance), Saint-Maurice, France

## Abstract

**Background:**

Nursing home residents bear a substantial burden of influenza morbidity and mortality. Vaccination of residents and healthcare workers (HCWs) is the main strategy for prevention. Despite recommendations, influenza vaccination coverage among HCWs remains generally low.

**Methods:**

During the 2007-2008 influenza season, we conducted a nationwide survey to estimate influenza vaccination coverage of HCWs and residents in nursing homes for elderly people in France and to identify determinants of vaccination rates. Multivariate analysis were performed with a negative binomial regression.

**Results:**

Influenza vaccination coverage rates were 33.6% (95% CI: 31.9-35.4) for HCWs and 91% (95% CI: 90-92) for residents. Influenza vaccination uptake of HCWs varied by occupational category. Higher vaccination coverage was found in private elderly care residences, when free vaccination was offered (RR: 1.89, 1.35-2.64), in small nursing homes (RR: 1.54, 1.31-1.81) and when training sessions and staff meetings on influenza were organized (RR: 1.20, 1.11-1.29). The analysis by occupational category showed that some determinants were shared by all categories of professionals (type of nursing homes, organization of training and staff meetings on influenza). Higher influenza vaccination coverage was found when free vaccination was offered to recreational, cleaning, administrative staff, nurses and nurse assistants, but not for physicians.

**Conclusions:**

This nationwide study assessed for the first time the rate of influenza vaccination among residents and HCWs in nursing homes for elderly in France. Better communication on the current recommendations regarding influenza vaccination is needed to increase compliance of HCWs. Vaccination programmes should include free vaccination and education campaigns targeting in priority nurses and nurse assistants.

## Background

Influenza virus infection is a major public health problem as shown by its high morbidity [1,2] and mortality [3-5]. In nursing homes for elderly people, influenza outbreaks have often been documented. In these settings, higher rates of morbidity and mortality are observed in the elderly compared to those of their counterparts living in the open community [6].

Vaccination against influenza for nursing homes residents has proved to be effective in preventing respiratory illness, hospital admissions and deaths [7,8]. Vaccination of nursing home employees can also contribute to the prevention of influenza, the limitation of the use of health services, and the reduction of deaths from pneumonia and other causes in residents [9,10]. Currently, French recommendations for yearly seasonal vaccination include all persons over 64 years, nursing homes residents and professionals who are in contact with them. Despite widespread recommendations in industrialised countries, influenza vaccination coverage among health care workers (HCWs) of nursing homes remains generally low [8,10-12].

We conducted a nationwide study in France during the 2007-2008 season to estimate influenza vaccination coverage rates in HCWs and residents in nursing homes for elderly people, and to identify determinants of vaccination status in these settings.

## Methods

### Populations

There are two types of nursing homes for the elderly in France. On one hand, medico-social institutions which are divided into elderly care residences (ECR), apartments for seniors and temporary housing. Apartments for seniors and temporary housing were excluded from our study, because living conditions are very similar to the ones of the open community, where residents are less dependent and share less common activities. On the other hand, health care institutions are typically defined as long term care facilities for elderly (LTCF). The study therefore targeted ECRs and LTCFs for elderly in activity in France.

According to the French regulations, ethical approval was not required as this observational retrospective study was based on collection of aggregated data only. Participation in the study for nursing homes was voluntary.

### Sample size and randomisation

We performed a cross-sectional survey based on a one-stage stratified random sampling design. The sampling frame was the list of institutions recorded in a national database (FINESS), where every medico-social and healthcare institution in France is recorded. It includes 6,845 nursing homes (2,535 public ECRs, 3,441 private ECRs and 869 LTCFs).

The sample size was calculated to provide estimates of influenza vaccination coverage by type of nursing homes (ECR/LTCF) and by categories of staff (physicians/nurse and nurse assistants) with a precision of 5% (hypothesis: influenza vaccination coverage: 50%, design effect: 2 or 1 in ECRs for physicians, participation rate: 50% in ECRs, 30% in LTCFs, α risk: 5%). Small nursing homes were overrepresented in the sample in order to obtain accurate estimates of influenza vaccination coverage in these settings. Because the number of LTCFs in France was lower than the number expected for the study, all LTCFs were included in the sample. ECRs were stratified by status (public or private), size (3 strata) and geographical location. Due to a small number of nursing homes within a stratum, two strata were merged into a single one. LTCFs were all included in one stratum. In all, 48 strata were constituted.

The number of nursing homes included in the study amounted to 2,186 (561 public ECRs, 756 private ECRs and 869 LTCFs).

### Data collection

Data collection was conducted from February to March 2008 through a postal questionnaire. Questions were related to the administrative characteristics of the nursing home: type (ECR or LTCF), status, location, size (number of beds), presence of a medical coordinator, and a score which indicates the degree of dependency of residents in a nursing home. This score, called GMP for "Gir moyen pondéré" [13], is generated using a French national scale and is defined as follows: sum of individual score of each resident divided by the number of residents in the nursing home. Other questions were related to influenza vaccination during the 2007-2008 season. The data collected included the number of residents and staff members by occupational category in nursing homes during the study period, and the number of residents and staff immunized against influenza during the 2007-2008 season. HCWs were defined as any person employed in the nursing home regardless of his or her occupation. We also asked whether training or meetings on influenza vaccination were organized, and if free influenza vaccination was offered to HCWs (including both the vaccine and its administration) during the 2007-2008 season. For each nursing home, only aggregated data were collected. The questionnaire was filled-in either by the medical coordinator, the director or the administrative nurse of the facility. Responses were sent by regular mail or fax. One reminder was sent to all non-respondents.

### Data analysis

We performed a double data entry. The analysis was carried out by categories of HCWs which included medical staff (practitioners, nurses, nurse assistants) and non-medical staff (administrative staff, cleaning staff and recreational staff). In order to assess the determinants of influenza coverage, and because of the aggregation and overdispersion of data, we performed uni and multivariate analyses with a negative binomial regression [14]. We categorized the size of nursing homes in four groups (< 50 beds, 50 to < 100 beds, 100 to < 150 beds and 150 or more beds), the residents' dependency in two almost equal-sized groups (low if the median score of dependency of residents in the nursing home was ≤ 700, and high if the median score of dependency was > 700), and influenza vaccine coverage (IVC) of HCWs in four almost equal-sized groups: very low (≤ 15%), low (15% < IVC ≤ 30%), medium (30% < IVC ≤ 50%) and high (> 50%). All exposures with a p value < 0.2 in the univariate analysis were introduced in the multivariate model. Risk ratios (RR) and their 95% confidence intervals were used as measures of association. A p value ≤ .05 was considered as statistically significant.

Data analyses were performed using Stata 9.2^® ^(StataCorp, Texas, USA). Specific sampling weights were calculated for each stratum. All estimates were made using the "svy" command, which enables to take into account the sampling design and weights in all calculations (descriptive, confidence intervals, negative binomial regressions). Outcomes are given in percentages with their 95% confidence intervals (95% CI).

## Results

### Participation

Of the 2,186 questionnaires sent, 1,674 were completed (response rate 77%). Response rates were 80% for ECRs and 70% for LTCFs. A total of 205 questionnaires were excluded, because the record included either information on more than one institution or the data of an apartment for senior, or because the resident data was not limited to elderly people, but also included younger persons. Furthermore, 240 questionnaires were not analyzed due to the absence of data on the vaccination status. Therefore, the final sample was corrected for analysis and included: 1,218 nursing homes with 497 public ECRs, 377 private ECRs and 344 LTCFs. In all, 1,870 physicians, 8,174 nurses, 26,512 nurse assistants, 28,320 non-medical staff members, and 122,470 residents were included in the analysis.

### Characteristics of nursing homes

Ninety percent of nursing homes offered free influenza vaccination to their staff (Table 1). Free influenza vaccination was less frequently proposed in private than in public ECRs, and less frequently in public ECRs than in LTCFs. Training sessions or staff meetings on influenza and its vaccination were organised in 49% of nursing homes. Eighty-two percent of nursing homes reported the presence of a medical coordinator in the nursing home. This coordinator was more frequently present in private and public ECRs than in LTCFs.

**Table 1:** Estimates of nursing homes characteristics by category of nursing homes.

	% (95%CI)			
	
	All nursing homes	Private ECR	Public ECR	LTCF
**Organization**				
Presence of a medical coordinator	82 (80-84)	85 (81 - 88)	84 (80 - 86)	64 (59 - 69)
High dependency of residents	43 (40 - 46)	44 (40 - 49)	27 (23 - 31)	96 (93 - 98)
Free vaccination offered	90 (88 - 92)	84 (80 - 88)	95 (94 - 97)	99 (98 - 100)
Training or staff meeting organized	49 (46 - 52)	52 (48 - 57)	44 (39 - 48)	54 (49 - 59)

The degree of dependency of residents varied from one setting to the other: residents were more dependent in LTCFs than in private and public ECRs (high median score of dependency of residents for 96%, 44% and 27%, respectively).

### Influenza vaccination coverage among HCWs

Overall, vaccination coverage among HCWs was 36.0% (95% IC: 34.2 - 37.8) (Table 2). It was not significantly different between medical personnel (37.2%; 95% CI: 35.7 - 39.4) and non-medical staff (34.2%; 95% CI: 32.0 - 36.3). However, coverage for physicians (60.4%; 95% CI: 54.9 - 65.8) was significantly higher than for nurses (45.2%; 95% CI: 42.8 - 47.5) and nurse assistants (33.7%; 95% CI: 31.8 - 35.8). Among non-medical staff, influenza vaccination coverage was 33.5% (95% CI: 31.3 - 35.7) for cleaning staff, 40.8% (95% CI: 36.2 - 45.4) for recreational staff, and 34.1% (95% CI: 31.0 - 37.3) for administrative staff. The analysis by type of nursing home (Table 2) shows that, for all occupational categories, vaccination coverage was consistently higher in private ECRs than in both public ECRs and LTCFs.

**Table 2:** Influenza vaccination coverage of HCWs in nursing homes by occupation and category of nursing homes.

	Vaccination coverage % (95% CI)			
	
Occupational category	All nursing homes	Private ECR	Public ECR	LTCF
**All**	**36.0** **(34.2 **-**37.8)**	**45.2** **(41.4 **-**48.9)**	**29.8** **(27.8 **-**31.9)**	**30.1** **(28.0 **-**33.5)**
Physicians	60.4 (54.9 - 65.8)	73.3 (66.2 - 80.4)	50.1 (41.1 - 59.1)	60.3 (54.6 - 66.1)
Nurses	45.2 (42.8 - 47.5)	58.6 (53.7 - 63.5)	40.1 (37.0 - 43.1)	35.0 (31.6 - 38.3)
Nurse assistants	33.7 (31.8 - 35.6)	45.0 (41.3 - 48.7)	27.5 (25.1 - 29.9)	26.4 (23.8 - 29.1)
Non-medical staff	34.2 (32.0 - 36.3)	41.2 (36.7 - 45.7)	28.2 (25.9 - 30.5)	31.6 (27.4 - 35.7)

### Determinants of influenza vaccination coverage among HCWs

In the univariate analysis, working in a private ECR, a small nursing home, the possibility of a free delivery of influenza vaccination for HCWs, the organization of training sessions or staff meetings on influenza and its prevention through vaccination were associated with a higher influenza vaccination uptake among HCWs. All variables remained statistically significant in the final multivariate regression model (Table 3). Only significant variables in the univariate analysis are listed in the table. No significant association was found with the score of dependency of residents or with the presence of a medical coordinator in the nursing home.

**Table 3:** Influenza vaccination coverage of HCWs, RR and adjusted RR of the potential determinants.

Nursing homes characteristics	Vaccination coverage % (95% CI)	Risk ratio (95% CI)		
		
		Unadjusted univariate	Adjusted multivariate	*P *value
**Categories of nursing homes**				
Private ECR	45.2 (41.4 - 48.9)	1	1	
Public ECR	29.8 (27.8 - 31.9)	0.70 (0.64 - 0.76)	0.74 (0.68 - 0.81)	< 0.001
LTCF	30.1 (28.0 - 33.5)	0.66 (0.61 - 0.73)	0.67 (0.62 - 0.74)	< 0.001
**Size (number of beds)**				
150 or more	25.5 (21.7 - 29.4)	1	1	
100 to < 150	36.2 (32.7 - 39.7)	1.25 (1.04 - 1.50)	1.21 (1.03 - 1.42)	0.02
50 to < 100	39.6 (37.4 - 41.8)	1.38 (1.17 - 1.62)	1.35 (1.16 - 1.56)	< 0.001
< 50	48.1 (44.1 - 52.2)	1.63 (1.37 - 1.94)	1.54 (1.31 - 1.81)	< 0.001
**Free vaccination offered**				
No	17.0 (6.4 - 27.5)	1	1	
Yes	36.8 (35.1 - 38.4)	1.69 (1.20 - 2.37)	1.89 (1.35 - 2.64)	< 0.001
**Training or staff meeting organised**				
No	31.2 (28.6 - 33.7)	1	1	
Yes	40.7 (38.3 - 43.1)	1.28 (1.17 - 1.39)	1.20 (1.11 - 1.29)	< 0.001

The highest vaccination coverage of HCWs was observed in private elderly care residences with < 50 beds, which offered free vaccination and organised training or staff meeting. Influenza vaccine coverage reached 58.0% (95%CI: 49.6% - 66.7%) in these nursing homes.

The analysis by occupational category showed that all determinants are not shared by all categories of professionals. The analysis restricted to nurses, nurse assistants and non-medical staff identified the same determinants. When free vaccination was offered, vaccination coverage was higher among nurses and nurse assistants (RR: 1.6; 95% CI: 1.09 - 2.40, p < 0.02) and for non-medical staff members (RR: 2.6; 95% CI: 1.60 - 4.22, p < 0.001). When training sessions or staff meetings were organized, vaccine uptake increased among nurses and nurse assistants (RR: 1.2, 95% CI: 1.11 - 1.32, p < 0.001) and non-medical staff members (RR: 1.2, 95% CI: 1.07 - 1.30, p < 0.001). The analysis restricted to physicians showed that working in a private nursing home and the provision of training sessions or staff meetings (RR: 1.13, 95% CI: 1.0 - 1.28, p = 0.05) increased vaccination rates. However, no association with the size of the nursing home or the offer of free vaccination (RR: 1.11, 95% CI: 0.81 - 1.51, p = 0.52) was found following the multivariate analysis.

### Influenza vaccination coverage and determinants for residents

Vaccination coverage in nursing homes residents was 91.4% (95% CI: 90.4 - 92.2). Univariate and multivariate analyses showed that living in a LTCF, a high score of dependency of residents, and a high influenza vaccination coverage among HCWs (IVC > 50%) were predictive of a greater vaccination uptake among residents (Table 4). In contrast with the results obtained for HCWs, the size of the nursing home, the offer of free vaccination for HCWs and the organization of training or staff meetings had no impact on influenza vaccination coverage of residents.

**Table 4:** Influenza vaccination coverage of nursing homes residents, RR and adjusted RR of the potential determinants.

Nursing homes characteristics	Vaccination coverage (%95 CI)	Risk ratio (95% CI)		
		
		Unadjusted univariate	Adjusted multivariate	*P *value
**Categories of nursing homes**				
Private ECR	91.1 (89.6 - 92.7)	1	1	
Public ECR	90.7 (89.3 - 92.1)	1.0 (0.98 - 1.02)	1.0 (0.98 - 1.03)	0.84
LTCF	94.8 (93.8 - 95.7)	1.04 (1.02 - 1.06)	1.02 (1.00 - 1.04)	0.05
**Size (number of beds)**				
150 or more	92.5 (91.2 - 93.8)	1	1	
100 to < 150	90.8 (89.8 - 91.8)	1.00 (0.97 - 1.03)	0.97 (0.94 - 1.01)	0.16
50 to < 100	90.5 (88.5 - 92.6)	0.98 (0.95 - 1.01)	0.98 (0.95 - 1.01)	0.27
< 50	92.4 (90.2 - 94.7)	0.97 (0.94 - 1.01)	0.99 (0.96 - 1.03)	0.93
**Medical coordinator**				
No	92.3 (90.9 - 93.7)	1	1	
Yes	91.2 (90.1 - 92.2)	0.99 (0.97 - 1.00)	0.99 (0.97 - 1.01)	0.2
**Dependency of residents**				
Low	89.8 (88.4 - 91.3)	1	1	
High	93.3 (92.3 - 94.4)	1.03 (1.02 - 1.05)	1.03 (1.01 - 1.05)	0.002
**Influenza vaccination coverage among HCWs**				
IVC ≤ 15%	91.9 (90.3 - 93.4)	1	1	
15% < IVC ≤ 30%	90.6 (89.1 - 92.2)	0.99 (0.96 - 1.02)	0.99 (0.96 - 1.01)	0.26
30% < IVC ≤ 50%	90.4 (87.4 - 93.4)	0.99 (0.96 - 1.02)	0.99 (0.96 - 1.02)	0.65
IVC > 50%	93.4 (92.1 - 94.8)	1.02 (1.0 - 1.04)	1.02 (1.00 - 1.05)	0.04

## Discussion

Our study showed that the coverage of influenza vaccination is high among residents of nursing homes for elderly people in France, whereas HCWs influenza vaccination coverage is insufficient. HCWs represent various occupational categories employed in nursing homes, including medical and non-medical personnel with various levels of contact with the residents. Our study shows that the staff involved in direct patient care **are **not necessarily better immunised than those with no such direct contact. Although no differences were observed between medical and non-medical staff, influenza vaccination uptake varied by category within the medical staff. Consistent with the results of other studies, our analysis indicates that influenza vaccination coverage was higher among physicians than among other HCWs occupations [15-19].

The high influenza vaccination uptake observed among residents indicates that recommendations for residents are well followed. In France, influenza vaccination is provided free of charge for all persons over 64 years in France. They receive a personal voucher for a free vaccine from the national health insurance fund. However, despite this high vaccination coverage among residents, influenza outbreaks still occur in nursing homes even when the adequacy between the circulating strain and the vaccine **is **good [20,21]. The introduction of the virus, its dissemination through insufficiently vaccinated HCWs and intensive contacts with incompletely protected residents all contribute to transmitting influenza in nursing homes. Our study showed that despite the existing recommendations, influenza vaccine uptake among HCWs is insufficient in France, an observation shared by many countries. Influenza vaccination coverage of HCWs working in institutions varied usually from less than 10% [22,10], to around 40% - 50% [16,17], and rarely exceeded 50% [15,23]. Even at relatively low level (43% - 51%), influenza vaccination of HCWs have an impact on morbidity and mortality among the residents in these nursing homes [9,10].

Our study documents several pieces of information that will be useful to improve influenza vaccination uptake of HCWs working in nursing homes. Vaccination campaigns are relatively well followed by physicians, but seem to insufficiently overcome reluctance among nurses, nurse assistants and non-medical staff. One pragmatic approach would be to target and adapt the information given to the staff involved in direct patient care first: nurses and nurse assistants. As our study shows that information and education as key factors associated with influenza vaccination coverage, specific training and staff meetings targeting these categories (i.e. using adapted material) should be widely pushed forward.

According to our study, only half of nursing homes propose these types of measures in France. Educational campaigns could include staff in-service sessions, the use of posters or leaflets, mailings, and the organization of conferences. Furthermore, specific information should be given about the lack of proven efficacy of homeopathy. Homeopathic medications are widely used in France, and a study conducted in a French geriatric hospital reported that almost 60% of unvaccinated HCWs believed that "homeopathic medications are more effective than vaccination in preventing influenza" [19].

Our study also highlighted the role of easy access to free vaccination. Only 20% of HCWs were vaccinated when free vaccination was not offered. However, because 90% of the nursing homes already propose free vaccination, improvements which could result from this measure are likely to be low. Specific information should be given in LTCFs attended by residents with a high degree of dependency. In these settings, the vaccination policy for residents is particularly well followed, but a low vaccination coverage is observed among HCWs despite the fact that they are offered free vaccination in almost all LTCFs.

International studies have shown that a significant increase of vaccination coverage can be observed among all occupational groups when free vaccination is combined with a communication strategy [10,16,17,24]. But, influenza vaccination coverage among HCWs hardly reached more than 50%, and improving this rate will be challenging [23]. Comparatively, influenza vaccination coverage in the general population was 24% in 2005/2006 [25].

The role of the private status of nursing homes on influenza coverage of HCW needs to be explored further. Private nursing homes may have more encouraging policies for vaccinating their staff than public ones so that absenteeism is decreased during the influenza season. The role of the size of the nursing homes is unclear, but several reasons may explain these findings. One possible reason may be that nursing homes are more committed to their staff's health, especially when staff members are not numerous. Acquisition of influenza by HCWs may cause absenteeism, and the possibilities of a small team compensating for absenteeism are limited. Furthermore, one study showed that the fact of "believing that most colleagues had been vaccinated" was a main factor associated with complying with vaccination [26].

Our study has some limitations. First, we only collected aggregated data. Some individual information such as demographic characteristics, factors influencing the acceptance of the vaccine, knowledge on the influenza vaccine could not be collected. On the other hand, because the questionnaire was short and easy to complete, a good response rate was achieved. Secondly the questionnaire was self administered by senior staff in the institution and the accuracy could not be checked by the investigator, which could have lead to errors in responses. Thirdly, vaccination rates only included data reported by the nursing homes. That may possibly underestimate influenza coverage, especially among physicians who frequently do not work full-time in the nursing home, and could already be vaccinated outside the nursing home. Lastly, a recall bias can not be excluded, even if it was expected to be limited, because of the short time interval between the period of vaccination and the study. We believe, however, that the impact of these possible biases is likely to be limited. For instance, the estimates of influenza vaccination coverage obtained through this study are close to those observed in another study [21]. In this study, among 64 LRTI outbreaks that occurred in nursing homes and that were reported to the French institute for Public Health Surveillance (InVS) during the 2006-2007 season, the average influenza vaccine uptake was 38% among the staff and 91% among the residents. In another study, the influenza vaccine uptake observed among patients in geriatric health care settings was 88% during the 2002-2003 season [27].

## Conclusions

This nationwide study assessed for the first time the rate of influenza vaccination among residents and HCWs in nursing homes in France. The analysis of the determinants of influenza vaccination gives helpful insight for improving vaccination rates among categories of staff with the lowest influenza vaccine coverage. Vaccination programmes should include free vaccination and education campaigns targeting in priority nurses and nurse assistants who work in LTCFs.

## Competing interests

The authors declare that they have no competing interests.

## Authors' contributions

SV, DN, LF, JPG, DLB conceived the study. SV, DN, LF analyzed the results in consultation with JPG and DLB. DN performed the data collection. SV wrote the draft version and revisions of the manuscript according to the contribution of DN, LF, JPG, DLB. DLB have given final approval of the version to be published. All authors read and approved the final version of the manuscript.

## Pre-publication history

The pre-publication history for this paper can be accessed here:

http://www.biomedcentral.com/1471-2458/10/159/prepub
